# Application of Biomarkers in Cancer Risk Management: Evaluation from Stochastic Clonal Evolutionary and Dynamic System Optimization Points of View

**DOI:** 10.1371/journal.pcbi.1001087

**Published:** 2011-02-24

**Authors:** Xiaohong Li, Patricia L. Blount, Thomas L. Vaughan, Brian J. Reid

**Affiliations:** 1 Division of Public Health Sciences, Fred Hutchinson Cancer Research Center, University of Washington, Seattle, Washington, United States of America; 2 Department of Medicine, University of Washington, Seattle, Washington, United States of America; 3 Department of Epidemiology, University of Washington, Seattle, Washington, United States of America; 4 Division of Human Biology, Fred Hutchinson Cancer Research Center, Seattle, Washington, United States of America; 5 Department of Genome Sciences, University of Washington, Seattle, Washington, United States of America; University of Texas at Austin, United States of America

## Abstract

Aside from primary prevention, early detection remains the most effective way to decrease mortality associated with the majority of solid cancers. Previous cancer screening models are largely based on classification of at-risk populations into three conceptually defined groups (normal, cancer without symptoms, and cancer with symptoms). Unfortunately, this approach has achieved limited successes in reducing cancer mortality. With advances in molecular biology and genomic technologies, many candidate somatic genetic and epigenetic “biomarkers” have been identified as potential predictors of cancer risk. However, none have yet been validated as robust predictors of progression to cancer or shown to reduce cancer mortality. In this Perspective, we first define the necessary and sufficient conditions for precise prediction of future cancer development and early cancer detection within a simple physical model framework. We then evaluate cancer risk prediction and early detection from a dynamic clonal evolution point of view, examining the implications of dynamic clonal evolution of biomarkers and the application of clonal evolution for cancer risk management in clinical practice. Finally, we propose a framework to guide future collaborative research between mathematical modelers and biomarker researchers to design studies to investigate and model dynamic clonal evolution. This approach will allow optimization of available resources for cancer control and intervention timing based on molecular biomarkers in predicting cancer among various risk subsets that dynamically evolve over time.

## Introduction

Detection of cancer at an early stage could significantly reduce cancer mortality and the overall burden of cancer [1]–[4]. The most common cancer risk model is based on the classification of the population into three groups: (1) normal without cancer, (2) asymptomatic cancer (detectable and potentially curable), and (3) symptomatic cancer, [5], [6]. This model has provided a foundation for clinical approaches to cancer screening and early detection that have largely been based on tissue morphological features observed microscopically or via imaging. Recent advances in cancer biology and molecular technology now provide new opportunities to further refine cancer risk models for use in the clinic.

Inherited susceptibility and environmental exposures, including infectious agents, can modulate cancer risk in an individual over time, but dynamic interactions between these factors and evolving somatic genetic abnormalities that lead to cancer are poorly understood. With progress in molecular biology and genetics, it is widely believed that a panel of biomarkers assessing DNA, RNA, proteins, and/or metabolic processes can eliminate the shortcomings of morphologic diagnosis for early detection and cancer risk prediction. In fact, advances in technology have identified many molecular abnormalities that develop during neoplastic progression, some of which are highly associated with cancer [7]–[10]. Currently, there is a quest to find the perfect cancer biomarker(s) that could be used to separate “cancer” from “non-cancer”. Yet, thus far, no molecular biomarkers that significantly reduce cancer mortality with satisfactory sensitivity and specificity have become widely used in the clinic for early diagnosis or cancer risk prediction in the general population, although some genetic tests have been adopted for individuals with inherited susceptibilities to cancer [11]. The limited success in identifying robust biomarkers has been attributed to inadequate study designs or complexity of biospecimens [12], [13], biased biospecimens [14], and technologic [13], [15] and computational limitations [16]–[18]. Although all of these reasons contribute to the limited success of cancer biomarker development to some degree, a fundamental challenge to be considered for biomarker development is the dynamic, stochastic nature of clonal evolution.

In this paper, we first define cancer “risk prediction” and “early cancer detection” using two very simple physical systems. We then introduce the dynamic, clonal evolutionary concept as it relates to biomarkers in cancer development and review the challenges associated with the use of molecular markers in a clinical model. Finally, we present a general theoretical framework for using imperfect biomarkers for cancer risk stratification in the clinic, using a dynamic systems approach. The goal of this Perspective is to inspire an integrated approach to theoretical modeling and biomarker research in cancer risk management by incorporating a clonal dynamic point of view.

## Event Detection and Prediction in Deterministic and Stochastic Systems

**Figure 1:**
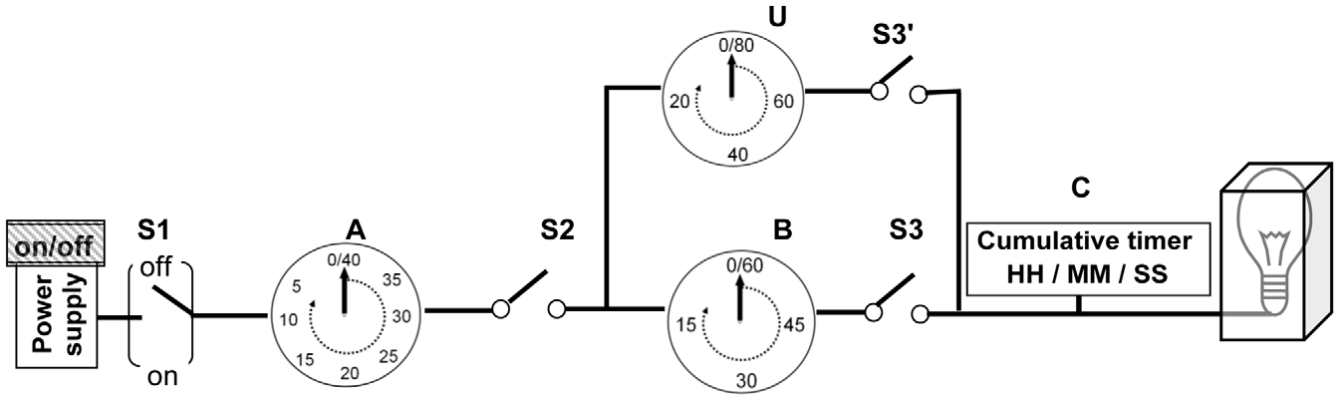
A simple deterministic physical system for illustrating event prediction and detection. In this system, an on-off switch, S1, is connected to a power supply and timer A. Timer A and B or U are each connected to a switch (S2, S3, or S3′) that will automatically connect the wire when the timers count down to 0. Switch S3 or S3′ is connected to cumulative timer C, which counts cumulative time since S3 or S3′ is switched on and which is connected to a light bulb that is situated in a metal box (whether the bulb is on or off is not observable directly). Timers A, B (and U), and C are set to their initial position at 40, 60 (and 80), and 0 seconds, respectively. Timer A starts to count down after S1 is set to the “on” position by turning power on. Timer A will count down 40 seconds, which will trigger S2 to switch to on position, which then triggers timer B and U to start counting down (pathway U is used to represent a possible alternative pathway). After timer B runs 60 seconds or U runs 80 seconds, it will set S3 (or S3′) to be connected, respectively, which will trigger timer C to count down and at the same time the light bulb will turn on. If one path (either B/S3 or U/S3′) is blocked, then the other one could still function to turn the light bulb on. Event prediction can be made with 100% certainty by observing timers A, U, or B before the light bulb turns on and event detection can be determined with equal accuracy by observing cumulative timer C after the light bulb is turned on.

**Figure 2:**
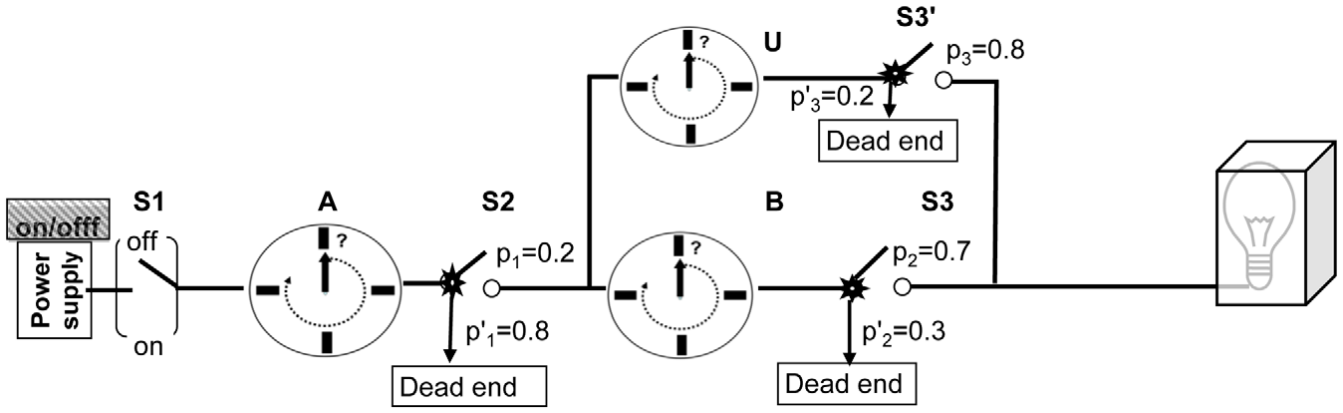
A simple stochastic physical system for illustrating event prediction and detection. Figure 2 is similar to Figure 1 except that switches S2 and S3 (or S3′) have probabilities of P'1 and P'2 (P'3) being switched to dead ends that will never lead to a pathway that causes the light bulb to turn on (pathway U is used to represent a possible alternative pathway). The timers (A, B, and U) are also of random nature. In this system, due to random nature of S2 and S3 (or S3′) and timers, one will never be able to determine with precise accuracy when the light bulb will turn on, or whether it will turn on at all.

In order to better illustrate the problems associated with event prediction and early detection, we will introduce two simple physical systems as shown in Figures 1 and 2. For the purposes of this illustration, we are interested in determining (1) when the light bulb will turn on (event prediction) or (2) whether the light bulb is already on (event detection).

### Event Prediction and Event Detection in a Deterministic System

We first investigate the question of event prediction in the deterministic system (for any given time point or initial state, the system will always produce the same outcome) as illustrated in Figure 1. In this system, all of the switches (S1, S2, S3, and S3′) have been precisely identified and characterized and the timers in the system (A, B, and U) are 100% accurate and clearly observable. In this deterministic model, the status of the light bulb can always be predicted perfectly by simply observing timers A, B, and U (see Figure 1 legend for details). Therefore, the necessary and sufficient conditions for 100% successful event prediction in this system are (1) all of the switches in the system are identified and characterized, and (2) the timers reflect the exact time (sufficient conditions) that has elapsed since any two switches have connected and are observable.

The question of whether the event has already occurred (light bulb is on) and the time elapsed after occurrence (event detection) can be answered in Figure 1 by simply observing cumulative timer C. Alternatively, it could be determined by observing the status of switch(es) S3, (S3′). Thus, the necessary and sufficient conditions for event detection can be determined by simply observing the status of the final switches or cumulative timer C.

### Event Prediction and Event Detection in a Stochastic System

A stochastic system that may not always result in the same outcome for any given time point or initial state is shown in Figure 2. In this system, one cannot make a prediction with 100% accuracy as to when the light bulb will turn on due to the stochastic nature of switches S2 and S3 (or S3′) and timers A, B, and U. In order to make a reasonably good prediction as to when the light bulb will turn on, one will need to periodically recheck the status of the switches.

The “switches” in Figures 1 and 2 may be viewed as similar to the abnormalities (e.g., DNA mutations/LOH/chromosome copy gain/loss) that develop during neoplastic progression, and the timers as the time that has elapsed between the genomic alterations. The event of turning on the light bulb may be intuitively analogous to the development of cancer. However, neither of these models addresses the complexity of neoplastic progression.

## Biological Complexity of Molecular Markers for Early Detection of Cancer and Cancer Risk Prediction

Environmental factors, inherited genetic susceptibility, and dynamic gene–gene and gene–environmental interactions can generate new somatic genetic or epigenetic alterations in human cells. Some altered cells continue to divide with a low rate of genetic or epigenetic alterations, largely maintaining genomic integrity, whereas others continue to develop new alterations, generating new variants on which selection can act. Several investigators have used genetic and genomic data to develop models of neoplastic progression. Nowell proposed that a single cell gains a selective genetic advantage over neighboring normal cells, rendering it “neoplastic” [19]. Genomic instability and stepwise selection of genetic variants lead to clonal expansions of viable lineages of daughter cells in somatic tissue and underlies solid tumor progression while most variant cells or clones do not evolve to cancer. Later, Fearon and Vogelstein [20] first proposed a sequence of key genetic events during the evolution of a normal colon cell to colon cancer. Barrett et al. [21] reported the evolution of neoplastic lineages in Barrett's esophagus using spatial and temporal data in individual patients, including ordering of early key genetic events, as well as tracing genetic lineages of viable clones that produced a cancer and clones that were not selected for progression to cancer. Based on molecular data and population cancer incidence data, a number of stochastic and mathematical models have been proposed to evaluate mechanisms of initiation and development of cancer [22]–[26]. In addition, a stochastic system dynamic modeling approach was utilized to study the mechanisms of cancer genesis and progression by using combined data from various molecular biology studies [27]. The stochastic properties of selected and nonselected chromosomal abnormalities in hyper-dynamic evolution was further illustrated by a cell population heterogeneity study in which the highest level of non-clonal chromosome aberrations was closely coupled with the strongest tumorigenicity [28].

**Figure 3:**
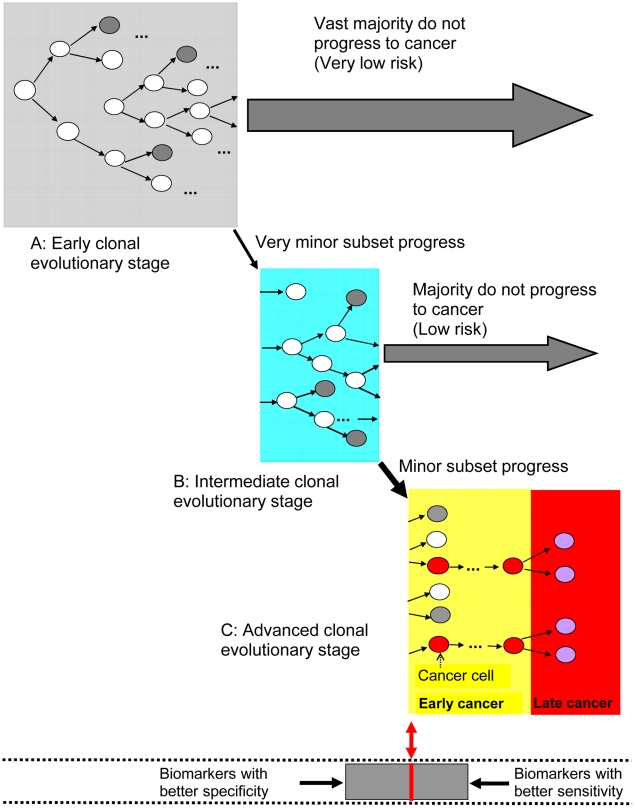
Neoplastic evolution, cancer risk prediction, and early cancer detection. (A), (B), and (C) represent evolutionary stages in dynamic clonal progression to cancer. (A) Early stages of clonal evolution have fewer selected genomic alterations, and most individuals do not progress to cancer. (B) A minority of individuals will evolve additional genomic alterations, but the majority of these will not progress to cancer. (C) A small subset of patients will accelerate development of genomic alterations leading to selection of increasing abnormal clones and progression to cancer. These events are stochastic and there are no biomarkers that perfectly distinguish (A), (B), and (C). In this evolutionary process, most clones may evolve in directions that do not lead to cancer (dark gray circles), whereas some others retain great potential for future progression to cancer or development of resistance to interventions to prevent or treat cancer (white circles), depending on selective pressures. Only a minority of the evolving clones will eventually acquire the capacity to become cancerous (red circles), and progression to cancer can occur by multiple possible pathways, also illustrated in the figure. The initial cancer cells may continue to divide locally and produce future metastases (purple circles in red block). The gray bar surrounded by dashed lines at the bottom of the figure illustrates the use of biomarkers in a clonal evolutionary system. Biomarkers with increasing specificity and sensitivity would shrink the gray block from either side toward the small center red bar, at the conceptual transition between non-cancer and cancer. A biomarker with perfect sensitivity and specificity would exactly correspond to the position of the red bar with perfect separation of cancer and non-cancer. Thus far, no biomarkers satisfy the necessary and sufficient conditions for precise cancer risk prediction or early cancer detection (as in Figure 1).

**Figure 4:**
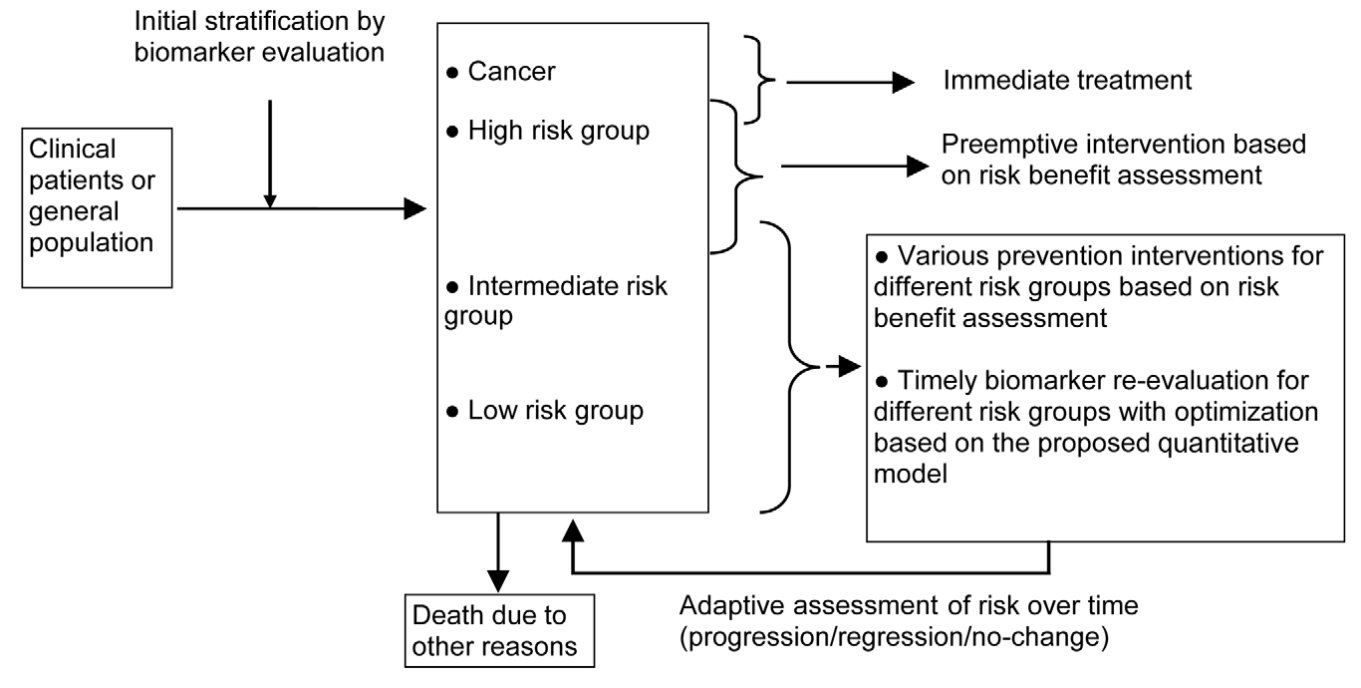
Cancer risk stratification and dynamic risk management using molecular biomarkers. Clinical patients or the general population could be stratified objectively using molecular biomarkers. A set of robust and validated biomarkers is expected to stratify the majority of patients into either high or low risk groups, and assign fewer patients to the intermediate risk group. The cancer risks of each group could be managed with consideration of individual risk, risk-benefit assessment, quality of life available resources, and dynamic progression characteristics of each group to achieve overall optimized results.

Many studies have shown that advanced epithelial malignancies have typically accumulated large numbers of genomic abnormalities not found in normal cells, including whole or segmental chromosome copy number amplifications, deletions, loss of heterozygosity, translocations, and point mutations [29]–[35]. Jones et al. [10] used spatial data from individual patients with colorectal cancers and reported that times between benign, invasive, and metastatic colon tumors can be estimated by analysis of the mutations they have in common and knowledge of the time it takes for cell division. Although these biological and genetic models provide significant insights for understanding evolution of a normal cell to cancer, all of them have stochastic characteristics (similar to Figure 2), and none of them meet the conditions required for perfect cancer risk prediction. This is because the exact steps and precise time elapsed between each step cannot be predicted with 100% accuracy in contrast to Figure 1. As a consequence, in clinical practice we will likely have imperfect biomarkers to identify high risk persons for targeted prevention strategies (cancer risk prediction) and those with early stage curable cancer for treatment (combination of cancer risk prediction and early detection). We next propose a schematic model that could be used for modeling cancer risk management with consideration of stochastic characteristics in the evolution of cancer (Figure 3) and more accurate risk stratification using current molecular measurements, as illustrated in Figure 4.

## Cancer Risk Prediction, Early Detection and Management: A Dynamic Systems Point of View

### Modeling the System

Previous models [5], [6], [36], [37] developed for cancer screening and early detection have been based on the definition of three conceptual stages: normal, cancer without symptoms, and cancer with symptoms. The analysis of screening for early detection of cancer was carried out using assumptions about the statistical distributions of transition from non-cancer to cancer and sojourn time. The approach of using three conceptual stages for classification of populations for modeling screening is reasonable. However, these models can not be easily related to, or characterized by, the complexity of clonal evolution as reported by recent genomic studies using high throughput sequencing or high density arrays.

One obvious approach is to use molecular or genomic biomarkers to stratify populations into various risk groups where each group has its own cancer risk distribution rather than treating overall cancer risk as a single distribution pattern in a population. However, molecular biomarkers are likely to be imperfect and have stochastic characteristics (Figure 3), and thus far, none of them meet the conditions required for perfect cancer risk prediction. In practice, one will be using imperfect biomarkers for both risk prediction and early detection. As shown in Figure 4, biomarkers could be used to stratify populations into different risk groups [38]–[43]. Each risk group could then be individually managed, with the goal of reducing the overall cancer risk over time, given a set of fixed resources or time constraints.

Our framework for cancer risk management considers the situation in which members in lower risk groups could progress to higher risk groups over time or, alternatively, members in higher risk groups could regress to a lower risk status. Such dynamic risk status will need to be periodically re-evaluated over time using biomarkers. Patients with cancer will be treated immediately, while a high risk group could be provided with an intervention to prevent cancer, such as chemoprevention, or monitored more closely for early detection. The lower risk groups could be subjected to much less intensive monitoring. Therefore, early detection and cancer risk management would comprehensively consider a dynamic system over a period of time that includes (1) the absolute risk for each risk group (cancer incidence rates), which is characterized by molecular biomarkers and estimated through population studies, (2) a pre-specified level of late stage cancer reduction, (3) resources available for cancer risk reduction or management, (4) the quality of an individual's life (effects of false positive or false negative diagnosis on quality of life), and (5) competing mortality due to other causes as shown in Figure 4.

There are many potential mathematical approaches to quantify or model such a dynamic system (Figure 4) for cancer risk management. In this Perspective, we suggest one possible method using dynamic system optimization to deal with clonal evolution for cancer risk management using biomarkers (Text S1).

## Discussion and Concluding Remarks

The development of cancer is a complex process characterized by stochastic accumulation of mutations and dynamic evolution of clones [10], [31], [44]–[46]. To date, most biomarkers are not directly related to evolutionary dynamics, but instead focus on specific pathways. However, extrapolating information from pathways to evolutionary dynamics remains a challenge [47]. Recently, genome-based cell population heterogeneity rather than commonly shared pathways have been linked to tumorigenicity [28]. Whole genome instability measurements are also closely linked to stages of cancer progression [29]. This body of evidence indicates that useful biomarkers can be developed directly using longitudinal measurements of genomic instability.

Development of biomarkers for cancer risk management should consider these stochastic and dynamic properties over time in neoplastic evolution. Using biomarkers for cancer risk management involves multi-level systems from cells to individuals to populations. Better ways of modeling multi-level systems and comprehending innate uncertainties in these systems are areas in which great benefits could be achieved [48]. We propose a dynamic system optimization approach to deal with the practical limitations of earlier three-stage models (Figure 4 and Text S1).

The goal of this Perspective is to link the dynamic, stochastic elements of clonal evolution in neoplastic tissues in patients followed over time. This would be best accomplished by collaborations between mathematical modelers and laboratory researchers in longitudinal experimental design, modeling, and parameter estimation oriented to practical application for cancer risk management.

There are fundamental differences between our concept and the commonly used three-stage (normal, cancer without symptoms, and cancer with symptoms) model for cancer screening: (1) we evaluate cancer risk prediction and early detection from a dynamic clonal evolutionary point of view and its implication for cancer risk management in clinical practice; (2) based on stochastic clonal evolution, we propose a framework to guide future biomarker research to more accurately stratify patients into various risk groups, where each risk group has a different cancer risk distribution, thereby permitting an adaptive cancer risk strategy; and (3) we propose that mathematical models be developed for cancer risk management that can be expanded for modeling specific cancer or management aspects, allowing optimization of available resources and intervention timing based on particular biomarker sensitivity and specificity in predicting cancer among various risk groups that dynamically evolve over time (Text S1). In addition, points (2) and (3) above take into account clonal dynamics in cancer development, including both progression and regression depending on dynamic selective conditions. Models using biomarkers for cancer risk management should allow optimization of various specific and objective functions dynamically, which is different from the traditional medical decision tree approach. Note that we addressed intrinsic stochastic properties of clonal dynamics in cancer development, as distinct from issues such as noise due to measurement error in biomarker studies.

In summary, effective prevention and early diagnosis strategies are critical to reducing the cancer burden. We analyzed the biological basis for using biomarkers for cancer risk prediction and early detection. This analysis shows that if and only if biological pathways for cancer development are fully determined and quantified as in Figure 1, can perfect accuracy for cancer risk prediction and early detection be achieved. With the advances of molecular technology and knowledge, biomarkers could reach a high level of accuracy in cancer risk prediction and early detection and could be used to guide clinical monitoring and interventions but likely will never be perfectly accurate. We propose that evaluation of biomarker effectiveness for cancer risk prediction and early detection be conducted with consideration of cancer evolutionary dynamics and dynamic optimization modeling for risk management.

## Supporting Information

Text S1 A dynamic optimization approach for cancer risk management and early detection. In S1, we present a framework of mathematical modeling for cancer risk management using biomarkers with consideration of stochastic clonal evolution in neoplastic progression.
(0.03 MB DOC)
