# Supplementary material for: Application of Biomarkers in Cancer Risk Management: Evaluation from Stochastic Clonal Evolutionary and Dynamic System Optimization Points of View
Source: PLoS Comput Biol. 2011 Feb 24;7(2):e1001087. doi: 10.1371/journal.pcbi.1001087 (PMC3044766; doi:10.1371/journal.pcbi.1001087)
Supplement: Text S1 — A dynamic optimization approach for cancer risk management and early detection. In S1, we present a framework of mathematical modeling for cancer risk management using biomarkers with consideration of stochastic clonal evolution in neoplastic progression. (0.03 MB DOC) [file pcbi.1001087.s001.doc]

**Text S1**

**A dynamic optimization approach for cancer risk management and early detection.**

Cancer development is a dynamic process. Therefore, the following differential equation could be used to model the dynamics of cancer development and cancer risk management in various risk groups over time as shown in Figure 4.

$dX/dt=f(X(t),Se_{i},Sp_{i,}u_{i}(t),\mathop{}\limits t)$, where X(t) =[*x_1_(t)*, *x_2_(t), x_3_(t), … x_i_(t)*] denoting the number of patients in the *i*th risk groups, which could dynamically either progress from lower to higher risk groups, or progress to cancer with different rates; or regress from higher risk group to lower risk group due to dynamic stochastic evolution; *Se_i_* and *Sp_i_* are the sensitivity and specificity of biomarker(s) when it is used for cancer risk management in *i-*th risk group (if the marker performed equally well for cancer risk stratification in the various risk groups, then *Se_i_* and *Sp_i_* are all equal); *u_i_(t)* denoting the efforts or costs of cancer risk management applied to *i*th risk group. X(t_0_)=X_0_ represents boundary condition (initial status of various risk groups at time 0) at the beginning time of the cancer management system. The goal is to properly manage each risk group to drive the *Xt* to targeted value *Xt_f_* in time *t_f_* , minimizing the following cost function *J*:

*J* = $\varphi[X(t_{f}),t_{f}]$ + $\int_{t_{0}}^{t_{f}} F[X(t),u_{i}(t),t]dt$, where $\varphi[X(t_{f})]$ is the final state penalty. The total cost of early cancer detection and cancer risk management is bounded by *u*: $\sum u_{i}\leq u$. Based on this dynamic quantification framework, different optimization plans could be implemented for different objectives for cancer risk management using biomarkers. For example, given fixed resources and a finite population at risk for cancer, one could use the model to determine the best use of resources and biomarkers to achieve the pre-specified reduction of late stage cancers without time limitation (*t_f_* is free in above). Alternatively, given fixed resources and a finite population at risk for cancer, one could use the model to determine the best use of resources and biomarkers to reduce the cancer incidence to a targeted level within a given time limitation (*X(t_f_)* and *t_f_* in above are both bounded). This model is very flexible and can be used for various cancer risk management scenarios using data collected in future studies.
